# Prenatal dispositions and genetic analysis of monozygotic female twins with suprasellar cysts and hydrocephalus: A case report

**DOI:** 10.1007/s00381-023-06245-z

**Published:** 2023-12-06

**Authors:** Frederikke Guldberg, Carl Christian Larsen, Elsebet Østergaard, Jonathan Carlsen, Marianne Juhler, Tina Nørgaard Munch

**Affiliations:** 1 Department of Neurosurgery, Copenhagen University Hospital, Copenhagen, Denmark; 2 Department of Clinical Genetics, Copenhagen University Hospital, Copenhagen, Denmark; 3 Department of Radiology, Copenhagen University Hospital, Copenhagen, Denmark; 4 Department of Clinical Medicine, University of Copenhagen, Copenhagen, Denmark; 5 Department of Epidemiology Research, Statens Serum Institute, Copenhagen, Denmark

**Keywords:** Suprasellar cyst, Twins, Obstructive hydrocephalus, Endoscopic fenestration, Prenatal dispositions

## Abstract

**Introduction:**

We present a unique case of monozygotic female twins with virtually identical clinical and radiological presentations of supratentorial hydrocephalus and cystic formations from the suprasellar cistern.

**Discussion:**

Evaluating genetic predispositions and prenatal exposures is crucial for hydrocephalus in twins. Familial cases imply a genetic contribution to the development of these anomalies, including chromosomal abnormalities and specific variants linked to arachnoid cyst formation in various syndromes. Extensive genetic analyses found no pathogenic variants in the twins. Prenatal exposure to anti-epileptic medication was known during pregnancy and may be associated with fetal abnormalities, but not central nervous system (CNS) malformations, and was therefore not considered the cause of the condition in the twins. The twins presenting simultaneously with hydrocephalus caused by suprasellar cysts (SAC) underwent a two-step surgical management: initial ventriculoperitoneal shunt (VPS) placement followed by fenestration. Postoperative imaging showed cyst reduction, but a secondary VPS was necessary in both cases.

**Conclusion:**

Genetic analysis is less likely to identify a monogenic etiology in non-syndromic cases of SACs, which are assumed to be multifactorial. There is no established evidence linking a teratogenic effect of anti-epileptic drugs to CNS malformations. Moreover, the surgical treatment of this complex condition constitutes a point of discussion.

## Introduction

Suprasellar arachnoid cysts (SAC) are benign, fluid-filled sacs evolving from the diencephalic Liliequist membrane as an extension of the dorsum sellae [1]. The incidence of arachnoid cysts is 1%, and out of this fraction, approximately 10% are suprasellar cysts [2]. Arachnoid cysts are often present at birth, and prenatal scans can demonstrate them as early as 20 weeks of gestational age [3]. While the etiology of SACs remains multifactorial, emerging evidence suggests a monogenic etiology in some cases for primary cysts. This case report presents a strikingly similar clinical and radiological manifestation of supratentorial hydrocephalus and cystic formations arising from the suprasellar cistern in monozygotic female twins.

## Case report

Monozygotic female twins were delivered by cesarean section in gestational week 34 + 1 in February 2018 due to breech position. The mother went into labor due to premature rupture of the membrane (PPROM). The twins’ mother suffers from epilepsy and experienced a few generalized tonic–clonic seizures during the pregnancy. She was treated with the antiepileptic drugs topiramate and levetiracetam throughout the pregnancy. The twins had two older, healthy siblings, one of whom was a maternal half-sibling. Twin A weighed 2570 g and was 46 cm long, and twin B weighed 2132 g and was 45 cm long at birth. None of the twins had cranial abnormalities or distended fontanelles, and they had normal head circumferences of 31.5 cm and 31 cm, respectively. There were no familial predispositions for prematurity or hydrocephalus.

**Fig. 1:**
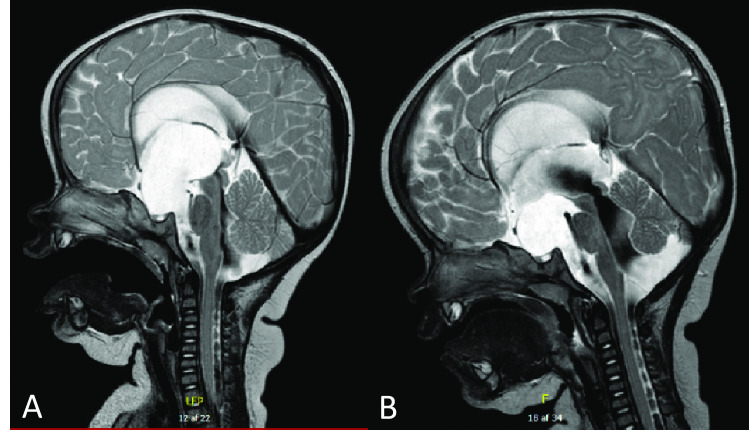
T2-weighted MRI scans at time of diagnosis for both twins **A** and **B** showing a suprasellar cyst indenting the third ventricle causing severe ventriculomegaly along with transependymal edema and basal cistern dilatation. Furthermore, but not illustrated on the pictures, both twins presented with supratentorial hydrocephalus with dilated lateral ventricles, temporal horns, and third ventricle

At 9 months old, the twins presented almost simultaneously with enlarging head circumference, bulging foreheads, distended fontanelles, and symptoms of regurgitation. In both twins, magnetic resonance imaging (MRI) demonstrated severe ventriculomegaly with transependymal edema (Fig. 1). A suprasellar cyst compressed pons, sulci, and the third ventricle, and stretching of the anterior visual pathways and corpus callosum. Furthermore, twin B’s MRI scan showed incomplete aqueductal stenosis. Both twins had Orbis Sigma II valve low pro (Integra Lifescience, Princeton, USA) shunts inserted, and no further shunt revisions were necessary. They displayed near normal development, with minor visual impairments and slight motor delays.

**Fig. 2:**
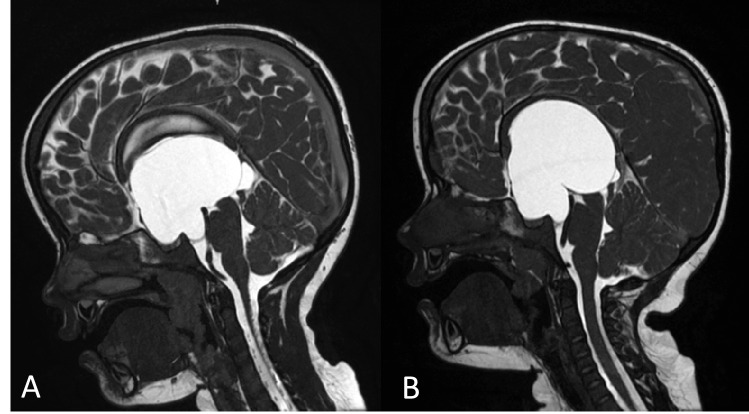
MRI CISS scans prior to fenestration: Twin **A**: expansion of the cyst measuring 64 × 58 × 65 mm and persistent ventriculomegaly. Twin **B**: expansion of the cyst measuring 61 × 37 × 56 with compression of the pons and persistent ventriculomegaly. Furthermore, but not illustrated on the pictures, for twin A, the large cyst extended through the foramen of Monro

At 4 years old, repeat MRIs due to delayed motor development revealed increased cyst size and aqueductal compression (Fig. 2). Both twins underwent ventriculocystocisternostomy and shunt removal, reducing cyst size on postoperative MRI. Subsequently, they developed ventriculomegaly with symptoms of increased intracranial pressure (headache, vomiting, and strabismus), presumably due to an absorption defect of the cerebrospinal fluid (CSF).

**Fig. 3:**
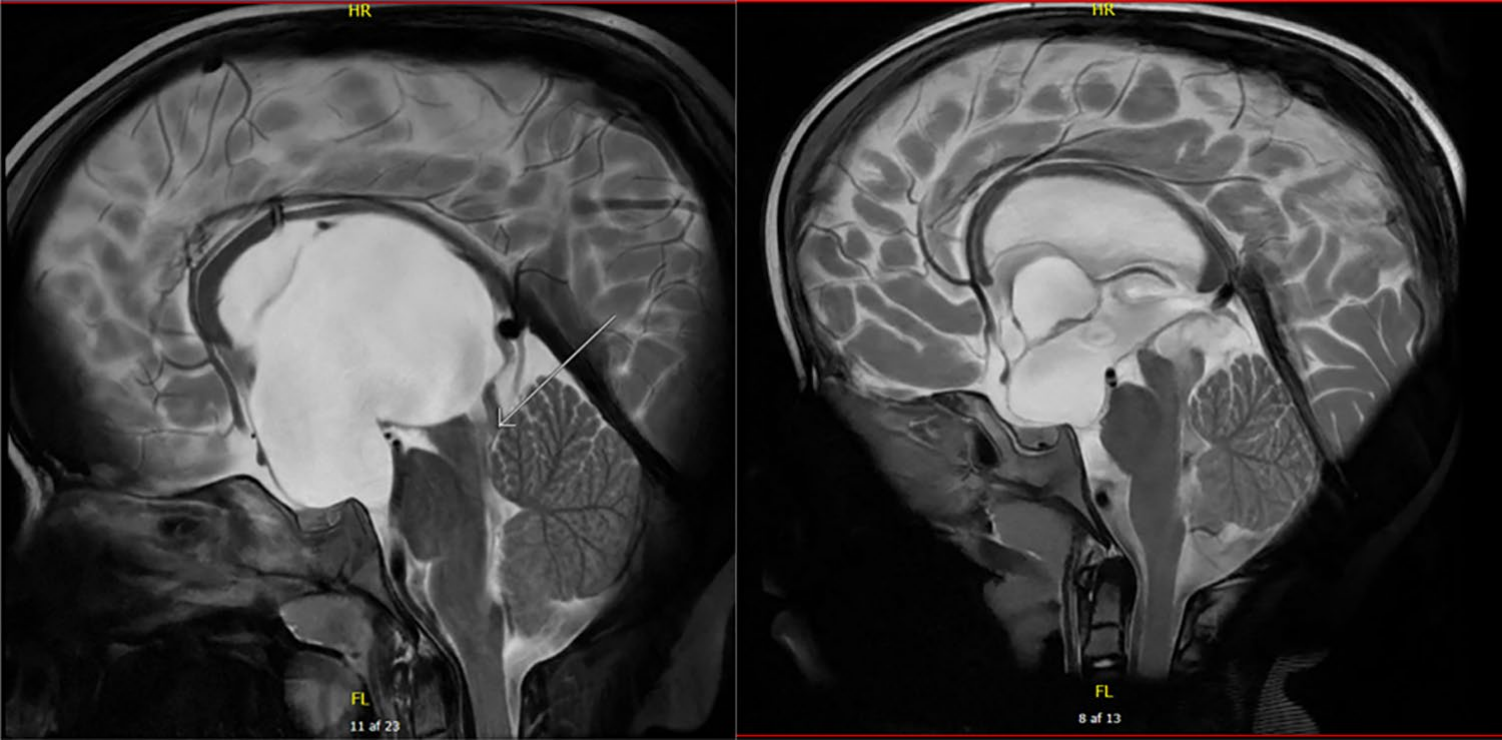
MRI flow scans after fenestration: Twin **A**: slight regression of ventriculomegaly and the suprasellar cyst measuring 54 × 46 × 60. Twin **B**: regression of suprasellar cyst and overall ventriculomegaly with the cyst measuring 53 × 23 × 21

Reinstating a VPS relieved symptoms in both twins, except for twin A’s strabismus. Postoperative MRI showed cyst size reduction and regression of the ventriculomegaly for both twins; however, patency of basal fenestration was unsure for twin A (Fig. 3).

Whole exome sequencing (WES) was performed on both twins and their parents [4] to search for variants in known disease-associated genes and in 121 candidate genes for hydrocephalus [5]. No variants were identified that could explain the twins’ phenotype.

## Discussion

This is the first report of virtually identical clinical and radiological presentations of supratentorial hydrocephalus and cystic formations from the suprasellar cistern in monozygotic twins. As reported in previous studies, the twins were initially treated with VPS during infancy [6, 7], followed by endoscopic fenestration beyond infancy [8, 9].

The genetic background of ACs is not well elucidated due to limited familial cases and complex pathogenesis. While suprasellar cysts are generally considered sporadic congenital defects, a few studies have identified familial cases spanning generations, supporting the significance of genetic factors [10–13].

### Genetics and inheritance

Studies of prenatal scans reveal that congenital ACs usually arise as midline cysts [14], often associated with anomalies causing ventriculomegaly and callosal abnormalities [15]. The presumed X-linked condition Aicardi, where the disease-causing gene is unknown, the X-linked dominant orofaciodigital syndrome caused by pathogenic variants in OFD1, and the autosomal recessive Chudley–McCullough syndrome with pathogenic GPSM2 gene variants often have interhemispheric ACs in addition to other symptoms [16–19]. Pathogenic FOXC2 gene variants cause lymphedema-distichiasis syndrome and have been associated with spinal extradural arachnoid cysts in several publications [10, 11, 13, 20–22]. Additionally, ACs have been linked to autosomal dominant polycystic kidney disease (ADPKD), particularly when caused by pathogenic variants in the PKD1 gene [23], and a familial case of posterior fossa AC and polycystic kidney disease has been reported [12]. In a family with six siblings with intracranial arachnoid cysts, suggesting autosomal recessive inheritance, linkage to the long arm of chromosome 6 has was found. However, to our knowledge, the disease-causing gene has not been identified [24].

Chromosomal abnormalities have also been linked to arachnoid cysts, including an unbalanced reciprocal translocation of chromosomes 14q and 20p [25], trisomy 20 mosaicism [26], trisomy 18 [15, 27], partial trisomy of 9q, and partial monosomy Xq [28]. These cases indicate genetic predispositions in AC development.

The WES analysis did not identify a monogenic cause for the twins’ SACs. Typically, in the literature, SACs appear in syndromic forms with associated symptoms and findings when a genetic background is identified. In these twins, we do not suspect a syndromic etiology. To our knowledge, no monogenic etiologies of isolated SACs have been reported; therefore, the likelihood of identifying a genetic background is low. Thus, the cause is most likely multifactorial with genetic and environmental factors shared between the twins. However, variants in unknown or unexpected genes linked to SACs cannot be entirely excluded.

### Prenatal exposure

Prenatal exposure to antiepileptic drugs increases the risk of congenital anomalies [29], e.g., fetuses exposed to topiramate have an increased risk of developing an oral cleft [30]. However, antiepileptic drugs are not established risk factors for cystic or other CNS malformations, so we do not believe that the ACs are a result of teratogenic effects of topiramate or levetiracetam.

## Conclusion

We have presented a rare case of suprasellar cysts causing hydrocephalus in monozygotic female twins. Further research is needed to elucidate potential causative genes associated with familial arachnoid cysts and to assess the role of other factors in the etiology of arachnoid cysts. Underlying causative factors should inform future tailored surgical management of complex hydrocephalus.
